# Association Between Habitual Food Intake and Energy Metabolism‐Related Urine Metabolites in Female Soccer Players

**DOI:** 10.1002/mnfr.70389

**Published:** 2026-02-10

**Authors:** Maria B. A. Nascimento, Maria M. S. Gouveia, Maryssa P. P. Dos Santos, Alessandre C. Crispim, Edmilson R. Da Rocha‐Júnior, Edson S. Bento, Thiago M. Aquino, Nassib B. Bueno, Filipe A. B. Sousa, Gustavo G. De araújo, Thays Ataide‐Silva

**Affiliations:** ^1^ Post‐Graduation Program in Nutrition, Faculty of Nutrition Federal University of Alagoas Maceió Brazil; ^2^ Laboraty of Applied Sports Science, Institute of Physical Education and Sports Federal University of Alagoas Maceió Brazil; ^3^ Nuclear Magnetic Resonance Research and Analysis Center, Institute of Chemistry and Biotechnology Federal University of Alagoas Maceió Brazil

**Keywords:** energy metabolism, food intake, metabolomics, NMR, women

## Abstract

To assess the association between habitual nutrients intake and the energetic metabolism‐related metabolites found by nuclear magnetic resonance (NMR) in female soccer players’ urine.Thirteen female soccer players were measured for habitual food intake 11 days before the competitive season, and had urine samples collected pre‐ and post‐match during five matches of two simultaneous championships to be examined by NMR. Nutrients and metabolites were associated through univariate and multivariate analyses. B3, B6, Prot.kg and energy (Kcal.kg) with leucine; B1, B3, B6, energy (Kcal.kg) and Prot.kg with AHI, glucose, tyrosine; formate and 1‐Methylnicotinamide. In parallel, inverse correlations were observed between B3, B6, and Prot.kg with creatinine, B12 and 1‐Methylnicotinamide (*r* ≥ 0.4; *p* < 0.05). In conclusion, it is possible to speculate that the herein described associations between metabolites and nutrients involved in energy metabolism seem to result from their participation in oxidative pathways, mainly in the TCA cycle and in gluconeogenesis.

## Graphical abstract

We studied the relationship between nutrient intake and substances found in urine, both involved in energy production, in female soccer players. We assessed the players' usual diet before the season and collected urine samples before and after matches. We found that some nutrients were associated with markers of energy production, including pathways that transform nutrients into fuel and produce glucose when more energy is needed during matches.

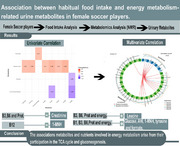

**Abbreviations**
2HI: 2‐hydroxyisovalerate
3AMI: 3‐aminoisobutyrate
3HI: 3‐hydroxyisovalerate
Acetyl‐CoA: acetil‐coenzyme A
AHI: alpha‐hydroxyisobutyrate
AQ: time acquisition time
BMR: basal metabolic rate
CHO: carbohydrate
D1: time between scans
EAR: estimated average requirement
EI: energy intake
ETC: electron transport chain
FAD: flavin adenine dinucleotide
FR: food record
LIP: lipides
LBM: lean body mass
MPM: multiple pass method
NMR: nuclear magnetic resonance
NS: number of scans
O1P: referring to water appearing irradiated
PLS‐DA: partial least‐squares discriminant analysis
Propynyl‐CoA: propynyl coenzyme A
Prot.: proteins
R24h: 24‐hour recall
Succinyl‐CoA: succinyl coenzyme A
SW: window width Signal
TCA cycle: tricarboxylic acid cycle
VIT: vitamin

## 1 Introduction

With a variety of fans and broadly spread around the world, soccer is among the most popular of all team sports [1], involving high‐intensity activities interspersed with submaximal periods during its matches [2, 3]. However, the matches’ scores are highly impacted by the individual athletes' capacity to perform such activities [4], which also influences their nutrient requirements [5]. Pertinent information about the athletes’ dietary pattern is essential to design accurate nutritional strategies, mainly when it comes to nutrients associated with energy metabolism, which are paramount to meet the required energy demands [6, 7].

Aerobic and anaerobic metabolic pathways provide the energy supply gathered during matches, and these processes involve macronutrients’ catabolism and body reserves’ degradation, respectively [8]. Vitamins belonging to the B complex play an essential role in acting as coenzyme components involved in the Krebs cycle, in macronutrients’ metabolism and in the constitution of molecules responsible for oxygen transport [9]. High‐carbohydrate diets lead to a large mobilization of glucose for energy supply, while low‐carbohydrate and high‐lipid diets can promote significant fat oxidation [10], often stimulating mitochondrial biogenesis to support enhanced oxidative capacity [11]. However, the intensity and duration of training, the level of training of individuals, the metabolic adaptations of athletes, and the intake of nutrients affect the mobilization of carbohydrates (CHO) and lipids for energy supply throughout exercise [10].

Regarding the nutrients responsible for providing energy, studies show that female soccer players do not seem to meet the recommended CHO intake [5, 12, 13], despite the fact that the physical effort required by an athlete during a soccer match can reach 75% of VO2Max, and maintaining energy supply depends on glycogen [14, 15]. In this sense, adequate CHO intake by soccer players helps maintain and recover glycogen in the body [15]. In general, the athletes have adequate lipid consumption [3, 6, 12]. Habitual food intake can be assessed through food frequency questionnaires or through the application of multiple 24‐hour recalls or food records (FRs). However, dietary assessment methods are subject to limitations such as portion size measurement, energy intake (EI) quantification, and respondent forgetfulness, which can result in an underestimation of food consumption. Metabolomic analysis, as it involves the quantification of metabolites in biological fluids, can help mitigate errors arising from dietary assessment methods [12, 13, 14], as indicated by recent studies [16, 17, 18].

Furthermore, this analysis can reflect the amount of food consumed, contributing to the development of precise strategies in the field of nutrition and not only presenting the variability of the response to diet, but also predicting such responses. In addition, urinary metabolomic analyses have the potential to help track food intake outcomes, as well as likely markers for the intake of certain food types and how an eating plan impacts the body [14, 16].

The dietary intake of female soccer players has been described in some previous studies [3, 5, 13, 19]. Although associations between self‐reported dietary intake and other variables, such as training load [3], somatic variables [19], inflammatory response and oxidative markers [13, 14, 15, 16, 17, 18, 19, 20, 21] have been analyzed, to the best of our knowledge, considerations about nutrient intake and its possible impact on professional female soccer players’ energy pathway‐related metabolomics expression have not been described, so far. Studies that use urinary metabolomic analysis applied to soccer generally aim to understand aspects related to variations associated with the physical and tactical aspects of sports performance [22, 23, 24, 25, 26, 27, 28, 29, 30], however, most of the time, they do not take into account nutritional aspects [26, 27, 28, 29] or do not present associations between metabolites and nutrients responsible for providing energy [30].

Glycine, formate, citrate, 3‐hydroxyvalerate (3HI), alpha‐hidroxyisobutirate (AHI) and glycolic acid are urinary metabolites related to energy metabolism in identified female soccer players after championship matches [30], but the possible influence of vitamins and nutrients involved in the expression of these compounds has not been described. Therefore, the objectives of this study were to evaluate the association between habitual nutrient intake and urinary metabolites in female soccer players involved in energy metabolism, as well as to describe and analyze the habitual dietary intake of the athletes. The hypothesis of the study is that there is an association between the change in the intermediate metabolites of energy pathways during soccer matches and the habitual CHO and lipids intake of female soccer athletes.

## 2 Experimental Section

### 2.1 Participants

Non‐probability sampling methods were applied to nineteen professional female soccer players from a state soccer team. They were invited to join the study after being informed about the possible risks and benefits of participating in it, prior to the commencement of the study. Subsequently, they signed the Informed Consent Form (ICF). This study is part of a larger project called “Analysis and Improvement of Athlete Performance” and it was approved by a Local Research Ethics Committee (n. 29269020.8.0000.5013), in compliance with ethical precepts in the Declaration of Helsinki.

### 2.2 Experimental Protocol

Athletes came to the laboratory only once before the championships to have their dietary data collected through one 24‐hour recall (R24h), and their anthropometric data. Also, habitual food intake was measured based on FRs through virtual collection 10 days before the championships (pre‐season period). Athletes filled in daily FRs and texted them in. They provided information on food, medication and dietary supplements through audio recording, text message and photographic reports. A critical information review was carried out with the athletes at the end of each FR collection, and the gathered information was subsequently included in the database. The multiple pass method (MPM) was adopted to reduce dietary surveys’ estimation errors of the R24h, we used the five steps of the MPM namely the quick list (everything consumed the previous day), the list of foods usually forgotten with definition of time and meal; the detailing and review (flavor, color, type, preparation method) and, finally, the final review of the information. [31]. Urine samples from each athlete were collected and placed in 25 milliliters (mL) containers at pre‐match and post‐match moments, over five consecutive matches. After collection, the samples were identified with the athlete's name and time of collection and stored in a cooler with dry ice at a temperature below −12°C and transported to the nuclear magnetic resonance (NMR) laboratory for further analysis, where they would be stored in a refrigerator at a temperature of −80°C. The athletes competed in two simultaneous championships in 2022.

### 2.3 Anthropometry

Weight and height were measured based on an anthropometric scale in a stadiometer (180 kg Adult Mechanical Scale, Welmy, Santa Bárbara do Oeste, Brazil); triceps, suprailiac and medial thigh skinfolds were measured with the aid of an adipometer (Lange Skinfold caliper, Cambridge Scientific Industries, Cambridge, United States). Athletes’ body fat rate was calculated through an equation based on three skinfolds for women [32].

### 2.4 Metabolomic Analysis

A 1.5 mL aliquot of the samples was transferred to Eppendorf tubes for sample preparation purposes. They were subsequently centrifuged at 14 000 rpm (MIKRO 220R) for 15 min, at 4°C. Then, 300 µL of supernatant from each sample was transferred to individual 5 mm NMR tubes, followed by the addition of 300 µL of 1 mM phosphate buffer solution (D_2_O, pH = 7.4, TSP = 1 mM). The analyzes were carried out on a BRUKER AVANCER spectrometer (Bruker, Karlsruhe, Germany), operating at 600 MHz for hydrogen analysis, using a 5 mm PABBO broadband probe using *noesygppr1d* pulse sequence to suppress the water signal through pre‐saturation—number of scans (NS) = 128, time between scans (D1) = 4.00 s, number of points in the spectrum of 64 thousand, window width (SW) = 20 parts per million (ppm), position where signal referring to water appearing irradiated (O1P) at 4.69 ppm and time acquisition time (AQ) = 5.11 s.

TopSpin software (version 3.6.5) (Bruker, Karlsruhe, Germany) was used for processing and viewing the spectra. Metabolites were identified based on the Human Metabolome Data base (HMDB) platform database (www.hmdb.ca) and Chenomx software (version 9.0) (Chenomx, Edmonton, Canada). To pre‐process the spectra (overlay, alignment and quantification of metabolites), the R software (version 4.2.2) (Lucent Technologies, Georgia, United States) was used, using the PepsNMR package (version 3.17). Finally a data matrix was obtained in .xls format, containing samples in rows and the quantification of metabolites in columns.

### 2.5 Habitual Food Intake Analysis

R24h and FRs, data from chemical composition tables [33, 34, 35, 36, 37] and home measurements [38] were tabulated in Microsoft Excel Software, version 2019. Subsequently, the dietary information was processed in the nutrition software Dietbox (Dietbox Informática, Rio Grande do Sul, Brazil), students’ version. B complex vitamins, EI (kilocalories) and macronutrients: CHO, Proteins (Prot.) and LIP (lipides %); as well as dietary fiber, were calculated. Data were exported to Excel, version 2019, by using Dietbox (Dietbox Informática, Rio Grande do Sul, Brazil), to create a database with the athletes self‐reported dietary information. Finally, the collected data were subjected to the double‐checking stage.

The energy, macronutrients [14] and dietary fiber [39] intake was assessed based on the literature on female soccer players. Micronutrients (B complex vitamins) were analyzed by using DRIS (dietary reference intakes) to assess individuals’ nutrient intake [40]. Calculations required by the method, as well as central tendency and dispersion measurements, were carried out in Jamovi software (version 2.2.5) (Jamovi Project, Sydney, Australia). Dietary surveys’ plausibility (FRs and R24h) was checked by taking into consideration the ratio between EI reported by the athletes and basal metabolic rate (BMR) calculated using the FAO/WHO/UNO prediction equation [41, 42, 43].

### 2.6 Statistical Analyses

Describes the central tendency (arithmetic mean and median) and dispersion (standard deviation) measurements, as well as the estimated average requirement (EAR) value recorded for B vitamins. Before performing metabolomic analyses, the data were processed using sum normalization, Pareto scaling, and logarithmic transformation using R Studio software (RStudio Team, 2020). Association between food intake variables and metabolites was investigated by combining two distinct correlation methods, firstly the Pearson's correlation coefficient was adopted to investigate all metabolite‐metabolite correlation and highlight the significant metabolite‐nutrient associations. Also, all correlation coefficients were tested for statistical significance based on a significance level of 5%. The correlation coefficients with p < 0.05 were considered different from zero and highlighted in the graphical representation. The adoption of an at least moderate Pearson's correlation coefficient (*r* value ≥ 0.4) [44]. In addition, a Data Integration Analysis for Biomarker Discovery using Latent Components model (DIABLO) [45], a data integration method derivate from the partial least‐squares (PLS) model, was employed to evaluate the association between metabolites and nutrients while considering the multivariate characteristics of the data. The model was built using both the athletes measured metabolites and the nutrient intake as predictor variables and the athletes identification across the matches as the categorical outcome. It's also noteworthy that this model considers both predictor datasets and generate a unified multivariate model containing the explained variability from the two blocks of variables, representing the shared variability between both datasets, while estimating the variables association and characterizing the profile of each individual athlete. Based on the DIABLO model, a similarity matrix analogous to the Pearson correlation coefficient was extracted and represented in the circus plot from Figure 2. The circus plot shows the associations between metabolites and nutritional intake variables estimated from the loadings on the DIABLO model components, moreover, to avoid overplotting and improve the readability, only the estimated correlations above 0.5 were represented, which also filters out correlations extracted from less representative components. All correlations were calculated using R Studio software (RStudio Team, 2020) with the packages Mixomics version 6.28 and corrplot version 0.92 [46].

## 3 Results

Travel to tournaments prevented some athletes from completing the data collection cycle, and only 14 participants completed the data collection phase. However, through dietary surveys’ plausibility analysis, we identified that one athlete had underestimated her food intake and therefore excluded her from the metabolic and dietary analyses. Thus, 13 (thirteen) athletes were eligible for analysis (age: 22.3 ± 3.06 years; height: 1.61 ± 0.07 meters; weight: 56.35 ± 6.10 kg; body fat: 19.02 ± 4.7%, and body mass index: 21.48 ± 1.52 kg/m^2^).

Habitual energy, nutrients, and fiber intake were measured before the state and national championships (Tables 1 and 2). According to the reports’ plausibility, 1 athlete under‐reported; 1 and 13 athletes accounted for plausible reports. Athletes recorded low mean CHO, Prot. and fiber intake. Habitual vitamins intake showed some probability of correctly concluding that food intake is adequate for 11 athletes (B1), 7 athletes (B2), 8 athletes (B3), 5 athletes (B6); 1 athlete (B9), and 8 athletes (B12).

**TABLE 1:** Habitual dietary intake of energy and macronutrients recorded for thirteen female soccer athletes during the pre‐season period.

Variables	Mean ± SD	Median	Minimum	Maximum	Recommendation	% mean adequacy
Energy intake (kcal)	1.367.5 ± 231.3	1.311.1	1098	1.980.5	—	—
Energy intake (kcal/kg of body weight)	24.46 ± 4.3	23.96	17.90	32.73	—	—
Energy intake (kcal/kg of LBM)	30.89 ± 6.66	28.40	24.35	48.60	30^a^	38.46
Prot. (g)	57.16 ± 16.67	59.20	34.53	89.97	—	—
Prot. (%)	16.60 ± 3.59	16.06	12.17	23.15	—	—
Prot. (g/kg of body weight)	1.03 ± 0.34	0.96	0.61	1.48	1.2^a^	46.15
CHO (g)	187.38 ± 35.06	193.39	129.18	257.84	—	—
CHO (g/kg of body weight)	3.32 ± 0.51	3.35	2.49	4.26	5–7^a^	0
CHO (%)	55.12 ± 8.30	55.47	41.85	65.99	—	
LIP (g)	43.26 ± 11.86	46.62	24.93	65.47	—	—
LIP (g/kg of body weight)	0.78 ± 0.24	0.83	0.36	1.08	—	—
LIP (%)	28.28 ± 5.28	29.75	18.16	35.10	20–30^a^	92.30
Fibers (g)	13.74 ± 4.51	14.10	8.72	25.18	25^b^	7.6

*Notes*: Reference value was not determined.

Abbreviations: CHO, carbohydrates; G, grams; Kcal, kilocalories; Kg, kilogram; LBM, lean body mass; LIP, lipids; Prot., protein.

^a^ [14].

^b^ [39].

**TABLE 2:** Habitual dietary intake of vitamins belonging to the B complex recorded for thirteen female soccer athletes during the pre‐season period.

Nutrients	Mean ± SD	Median	Minimum maximum	EAR value^a^
Thiamine‐B1 (mg)	1.12. ± 0.28	1.13	0.66–1.71	0.9
Riboflavin‐B2 (mg)	0.96 ± 0.35	0.94	0.39–1.75	0.9
Niacin‐B3 (mg)	16.02 ± 6.12	17.99	5.33–24.05	11
Pyroxidine‐B6 (mg)	0.99 ± 0.37	1.06	0.41–1.66	1.1
Folate‐B9 (mcg)	159.13 ± 80.13	149.20	49.89–380.6	320
Cobalamin‐B12 (mcg)	4.12 ± 6.65	2.50	0.87–26.06	2

Abbreviations: EAR, estimated average requirement; Mcg, micrograms; mg, milligrams.

^a^ [40].

**FIGURE 1:**
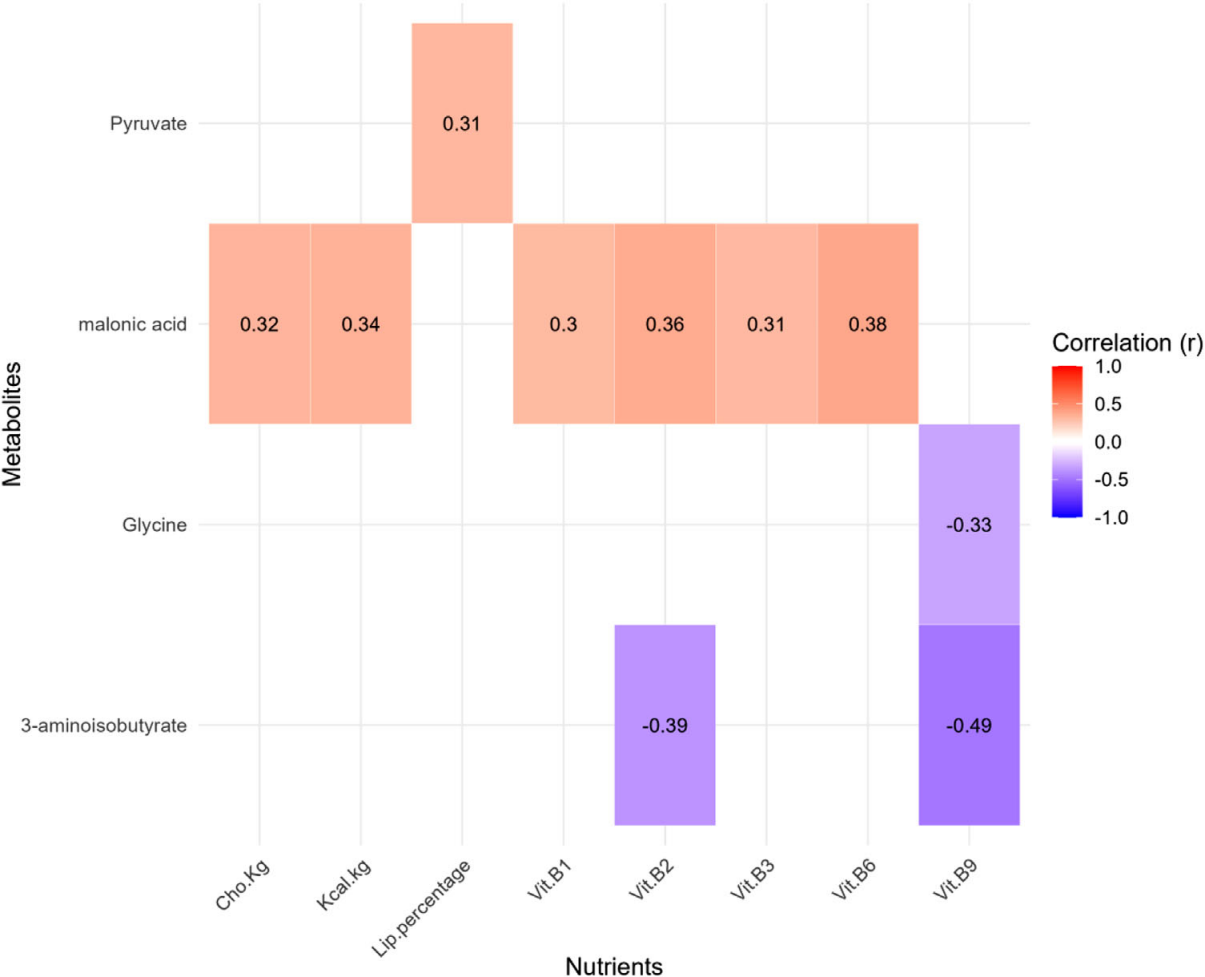
Pearson's correlation between habitual dietary of energy, macronutrients and B‐complex vitamins intake and urinary metabolites (*r* ≥ 0.4 and *p* ≤ 0.05) changes of professional female soccer players during championship matches. CHO, carbohydrate; PTN, protein; VIT, vitamin.

**FIGURE 2:**
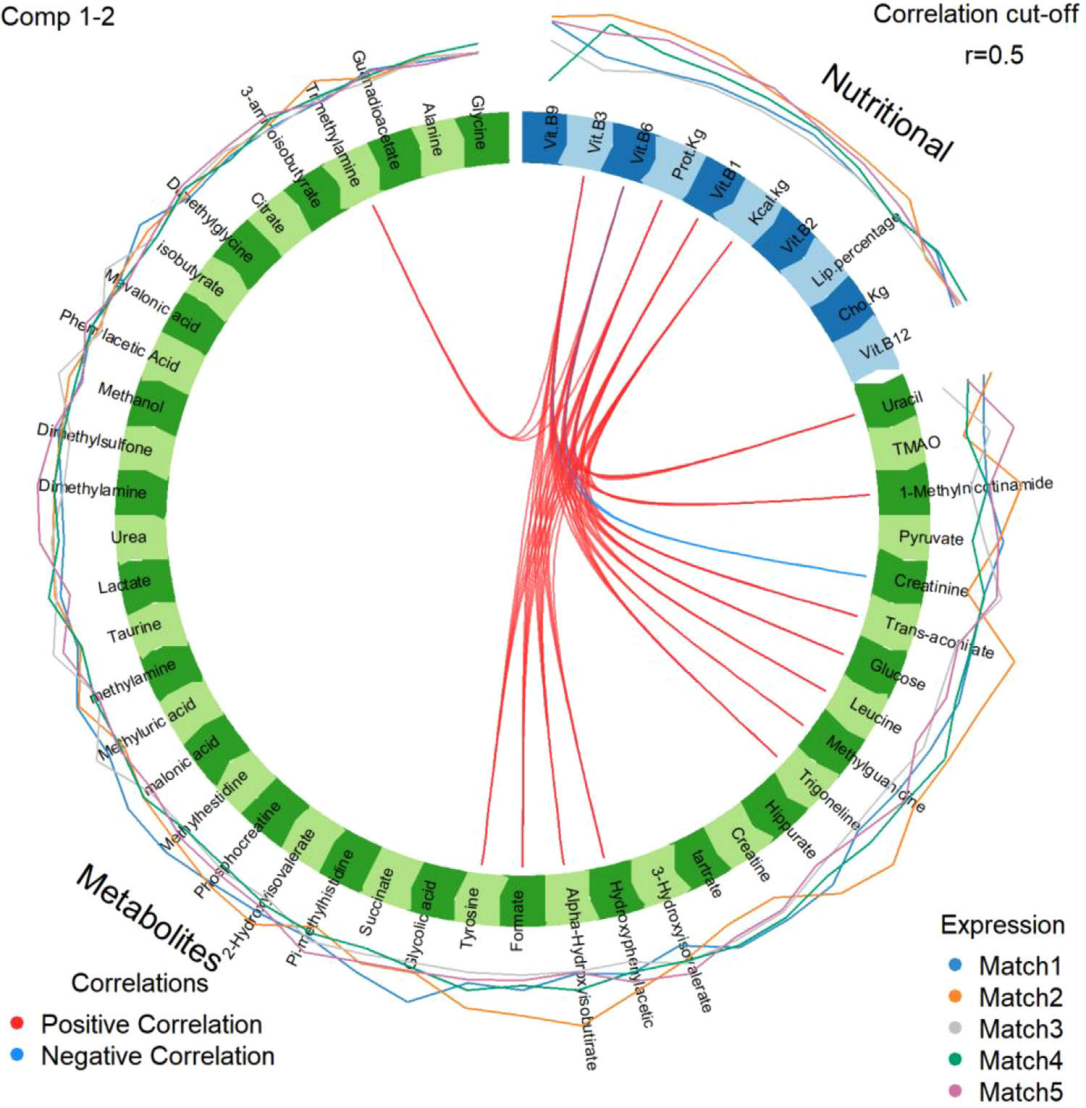
Multivariate correlation between habitual dietary energy, macronutrients and B‐complex vitamins intake and urinary metabolites of female soccer players over the championship matches. CHO, carbohydrate; PTN, protein; VIT, vitamin.

Citrate, lactate, glucose, pyruvate, succinate, AHI, 3HI, 2‐hydroxyisovalerate (2HI), formate, 1‐methylnicotinamide, alanine, tyrosine and leucine were the metabolites related to energy pathways identified in the samples. The association between mean nutrients’ intake and relative urinary metabolites’ during the matches is depicted in Figures 1 and 2. The R values are described in the image for Pearson's correlation and in the table for multivariate association (supplementary material 1).

The circular graph shows multivariate correlation between nutrients and metabolites (Figure 2). Lines outside the correlation circle show the mean expression of the group of variables set for each athlete over five matches between championships. The great variation in the external lines points towards greater expression of metabolites or nutrients in that match, whereas internal lines’ thickness points out the strength of correlation in a direct or inversely proportional association (Figure 2).

## 4 Discussion

Although we did not find associations between all the energy metabolites we hypothesized, we observed moderate correlations between nutrients and metabolites associated with the intermediate energy pathways of CHO and lipid metabolism, such as direct associations between B3, B6, Prot.kg, and energy (Kcal.kg) with leucine; B1, B3, B6, energy (Kcal.kg), and Prot.kg with AHI, glucose, tyrosine; format, and 1‐methylnicotinamide. In parallel, inverse correlations were observed between B3, B6, and Prot.kg with creatinine, B12 and 1‐Methylnicotinamide.In addition, the habitual food consumption for the herein assessed professional female soccer players showed low mean CHO, Prot.Kg and fiber intake. However, there was lipid intake adequacy among female soccer players [14]. Vitamins belonging to the B complex presented a low probability of adequate habitual food intake, mainly folic acid [40].

Thus, inadequate intake, particularly of vitamins B6, B9, and B12, may lead to decreased sports performance, while low folate levels may not only impair exercise efficiency but also compromise reproductive health and fertility in athletes [14]. The literature already describes female soccer players’ low intake of both energy and CHO [5, 19, 20, 47, 48]. Relative energy deficiency in sports increase muscle injuries, the number of injury reports is larger among women presenting menstrual period disorders than among athletes without menstrual impairment [49, 50]. The herein observed low CHO intake appears to have led to an energy imbalance in the assessed players' diet. We speculate that this intake may account for the increased expression of lipid metabolites such as AHI, 3HI, and 2HI, which are involved in energy provision [23]. These compounds were detected in the players’ urine after the match [23]. Although the athletes had an average EI (kcal/kg of LBM) in line with recommendations, only 38% had adequate consumption.

CHO intake values, in their turn, were below the recommended lowest limit set for female soccer players [14]. These values also seem to be closely related to dietary fiber intake results, since CHO rich foods, such as legumes, fruits, and vegetables, are important sources of dietary fiber. Other studies [5, 22] have also identified female soccer players’ dietary fiber intake below the recommended values [39]. However, the recorded values in the current study were even lower than the previously recorded. We believe that variations in competitive level may have influenced the discrepancies observed compared to previous studies [5, 21], however, there appears to be consistency in CHO intake among female soccer players. Inadequate CHO dietary intake favors muscle fatigue, and is known to produce negative effects on athletic performance [5, 6, 51].

Mean Prot. intake was not only below the advised values in recent recommendations [14], but also lower than results observed in other studies that have assessed professional female soccer players’ food intake [5, 49, 50]. Low Protein in athletes' diets can be detrimental to muscle Protein synthesis, body weight standardization and regeneration after matches and training, in addition, it can be a limiting factor for good performance [14]. Lipid intake reported by the assessed players was within values considered normal [14]. Although lipid intake among soccer players has been significantly decreasing over the last decades, most studies show lipid intake within the recommended values [3, 6, 21, 52] or even above values often recorded for sedentary individuals [5]. This outcome is in line with recommendations set for the general population [40].

Most athletes’ habitual nutrients intake measured here led to the conclusion that the intake of vitamins belonging to the B complex was inadequate. It is known that these nutrients act as cofactors in energy production and are related to exercise‐induced adaptations [26]. Thus, inadequate nutrient intake, mainly of vitamins B6, B9 and B12, can lead to decreased sports performance. Yet, low folic acid levels can pose risks to athletes’ health condition, in addition to impair efficiency during exercise [5].

Direct associations were observed among B3, B6, Prot.kg and energy (Kcal.kg) with leucine; B1, B3, B6, energy (Kcal.kg) and Prot.kg with AHI, glucose, tyrosine; formate and 1‐Methylnicotinamide. The association between grams of Prot. Kg and leucine, tyrosine, and glucose may be due to the degradation of amino acids in the glucose‐alanine cycle to provide energy to muscles in the form of glucose [53]. In this sense, we believe that this is the explanation for the association of Prot. Kg with the glucose metabolite, there is amino acids degradation in the glucose‐alanine cycle for energy production [53, 54, 55, 56]. However, it is worth noting that athletes recorded low CHO and EI in this study, given this possibility, the oxidation of amino acids for energy supply would be justified. The role of the glucose metabolite in the energy pathways of female soccer players was observed in a study [29].

Urine formate is described as part of CHO metabolism, however [57] and may be associated with a marker of fatigue in female soccer players [30] given the low CHO consumption and energy inadequacy, the association between Prot.kg and Energy (kcal.kg) with formate may be related to the mobilization of protein compounds and the oxidation of fatty acids to meet the high demands required by exercise [57]. With a scenario of high energy demand and possible inadequacy in the consumption of these nutrients, this association may reflect a strategic change in metabolism to meet the high energy demands throughout the matches. Accordingly, the correlation between vitamins of the B complex, mainly vitamin B6, with glucose reinforces speculations about the action of these compounds in energy pathways required during exercise.

Inverse correlations were observed between B3, B6, and Prot.kg with creatinine, B12 and 1‐Methylnicotinamide. The metabolite 1‐Methylniconamide is described as the end product of niacin, so the association between B3 and the metabolite may be related to the metabolism of the vitamin in the body [58]. The metabolite 1‐Methylniconamide is described as the end product of niacin, so the association between B3 and the metabolite may be related to the metabolism of the vitamin in the body. Because it results from the catalysis of NAD+ [57], which acts in the electron transport chain (ETC), promoting the activity of enzymes associated with fatty acid oxidation, we speculate that the association between compounds and energy pathways is related to lipid oxidation after exercise.

The action between 3‐AMI and a possible role in energy generation through glycide and lipid substrates is suggested in the literature [59], although associations between this metabolite and CHO and lipid intake were not observed in this study; we only observed correlations between 3‐AMI and B9. However, although we did not find associations between 3‐AMI and B6, the enzyme glyoxylate aminotransferase 2 (AGXT2), which acts in the conversion of the D‐Baiba enantiomer to D‐methylmalonate semialdehyde for subsequent oxidation to propinyl‐CoA, uses pyridoxal phosphate (B6) as a cofactor [60] Regarding energy generation, after oxidation to propinyl‐CoA, there is enzymatic conversion to succinyl‐CoA and utilization in the Krebs cycle through enzymatic conversion to succinyl‐CoA [59, 60].

To our knowledge, this study is a pioneer in carrying out an investigation that associates the energy metabolomic profile of professional soccer players with their usual dietary intake. However, this research also has limitations, such as the fact that confounding factors were not excluded from the study, which may explain the non‐significant correlations observed between metabolites and nutrients. We suggest that future studies consider the use of specific functional markers of vitamins and macronutrients in associations, and also investigate these associations at pre‐ and post‐matches. The results of self‐reported dietary surveys by athletes should be interpreted with caution and take into account that these methods are susceptible to underestimation or overestimation, regardless of whether the majority of reports are plausible. The pre‐season diet was taken into account, and the association between food intake during this period may not accurately reflect the dietary profile adopted during competition, however, it is possible to assume that during the short duration of the championships studied, the athletes' food intake did not change. Furthermore, the sampling methods adopted and the small sample size may limit the generalizability of the results, despite the fact that studies on the subject use similar sampling methods and sample sizes. Finally, we did not assess the impact of nutritional deficiencies on metabolic processes; therefore, future studies addressing this topic are encouraged.

### 4.1 Concluding Remarks

Macronutrients and dietary fibers’ habitual food intake by professional woman´s soccer players were below the recommended level, except for lipids. It has been observed a correlation between B3, B6, Prot.kg and energy (Kcal.kg) with leucine; B1, B3, B6, energy (Kcal.kg) and Prot.kg with AHI, glucose, tyrosine; formate and 1‐Methylnicotinamide. In parallel, inverse correlations were observed between B3, B6, and Prot.kg with creatinine, B12 and 1‐Methylnicotinamide. In conclusion, it is possible to speculate that the herein described associations between metabolites and nutrients involved in energy metabolism seem to result from their participation in oxidative pathways, mainly in the TCA cycle and in gluconeogenesis.

## Conflicts of Interest

The authors declare no conflicts of interest.

## Ethics Statement

The athletes were informed about all the procedures to be conducted during the research, as well as the possible risks and benefits, prior to the commencement of the study. Subsequently, they signed the Informed Consent Form (ICF). This study is part of a larger project called “Analysis and Improvement of Athlete Performance” and it was approved by the Research Ethics Committee (CEP) of the Federal University of Alagoas (UFAL) under the protocol number CAAE: 29269020.8.0000.5013 and Opinion: 4297907, in compliance with ethical precepts in the Declaration of Helsinki. The overarching project was supported by the National Council for Scientific and Technological Development (CNPq) under the code 408972/2021‐1.

## Data Availability

Data available on request due to privacy/ethical restrictions.
